# First South Korean Record of Beetle Suborder Myxophaga Extends Its Documented Range in Continental East Asia

**DOI:** 10.1002/ece3.74411

**Published:** 2026-09-22

**Authors:** Seunghyun Lee, Minhyeuk Lee, Wonwoong Kim

**Affiliations:** ^1^ Research Institute of Agriculture and Life Sciences, Seoul National University Seoul Republic of Korea; ^2^ Insect Biosystematics Laboratory, Department of Agricultural Biotechnology Seoul National University Seoul Republic of Korea; ^3^ National Institute of Agricultural Sciences Wanju Republic of Korea; ^4^ Department of Ecology and Evolutionary Biology University of Michigan Ann Arbor Michigan USA

**Keywords:** COI barcoding, first record, Korean peninsula, myxophaga, riparian microhabitat, *Sphaerius*, Sphaeriusidae

## Abstract

Myxophaga is a minute beetle suborder that is easily overlooked because of its small body size and specialization on wet microhabitats. Here, we report *Sphaerius* from two riparian localities in South Korea, representing the first record of Myxophaga from the Korean Peninsula. This discovery also represents the northeasternmost known record of Myxophaga from continental East Asia, distinct from previous island records in Japan and Taiwan. Specimens were collected from saturated riparian substrates near the active waterline. Scanning electron microscopy confirmed characters consistent with *Sphaerius*, and the Korean COI barcode sequences were distinct from all other available *Sphaerius* sequences, although their position within the genus remained unresolved. Because records of East Asian *Sphaerius* remain taxonomically undetermined, we identify the Korean material as *Sphaerius* sp. This record expands the known continental distribution of Myxophaga in East Asia and highlights the value of targeted surveys in riparian microhabitats.

## Graphical abstract

We report *Sphaerius* sp. from South Korea, representing the first record of Myxophaga from the Korean Peninsula and extending its known continental East Asian range northeastward. SEM‐based morphology and COI barcode data support its placement in *Sphaerius*.

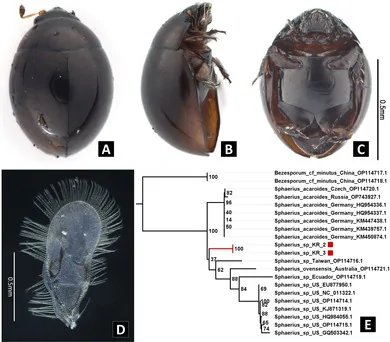

## 1 Introduction

Myxophaga is a small beetle suborder composed predominantly of aquatic and semiaquatic lineages. Its members are usually minute and occupy specialized wet microhabitats along the margins of streams, rivers, lakes, and hygropetric surfaces (Beutel 2005; Hájek et al. 2011; Beutel and Arce‐Pérez 2016; Fikáček et al. 2023). The Late Triassic fossil record of Myxophaga indicates that miniaturization and specialization for wet, algae‐rich habitats arose early in its evolution (Qvarnström et al. 2021; Fikáček et al. 2023). Because of their small body size and specialized habitat preferences, myxophagans are easily overlooked in conventional faunal surveys and may remain undetected even in relatively well‐studied regions (Fikáček et al. 2023). The suborder comprises four extant families, of which three, Sphaeriusidae, Torridincolidae, and Hydroscaphidae, are reported from East Asia.

The family Sphaeriusidae has historically been regarded as a small, nearly worldwide family, absent only from Antarctica. Until the revision of Fikáček et al. (2023), extant members were long placed in a single genus, *Sphaerius* Waltl, 1838 (Reichardt 1973; Löbl 1995; Hall 2019). Adults are generally 0.5–1.2 mm long and have a strongly convex, shiny, and dark brown to black body (Matthews 1899; Fikáček et al. 2023). Most known species live in saturated sand, fine gravel, mud, plant debris, or beneath wet stones close to the water's edge, although some terrestrial species are also known from moist leaf litter and other non‐riparian substrates (Löbl 1995; Kamezawa and Matsubara 2012; Liang and Jia 2018; Fikáček et al. 2023).

The Oriental Region has been considered especially species rich: Reichardt (1973) listed 18 known sphaeriusid species, eight of them from Southeast Asia, and additional terrestrial species were later described from South Asia (Löbl 1995; Liang and Jia 2018). In contrast, East Asian records remained sparse and taxonomically unresolved for a long time. Although Hall (2003) reported Sphaeriusidae from China and listed them as *Sphaerius* sp., the identity of this species, even its genus‐level assignment, remains in question (Liang and Jia 2018; Fikáček et al. 2023). Records from Honshu, Japan, were likewise left unidentified at species level (Kamezawa and Matsubara 2012; Saito 2018). The first named species from mainland East Asia was *Sphaerius minutus* Liang and Jia, 2018, described from Jinggangshan, Jiangxi Province, China, based on specimens collected beneath a wet stone at a stream margin (Liang and Jia 2018).

Recent work has substantially revised this framework. On the basis of scanning electron microscopy (SEM) and molecular phylogenetic data, Fikáček et al. (2023) recognized two living genera, *Sphaerius* and *Bezesporum*, and transferred *S. minutus* to *Bezesporum*. Their study showed that sphaeriusids are conservative in external morphology but include deeply divergent lineages, and further suggested that species‐level diversity in the family is likely underestimated. This is particularly relevant for East Asian material, because Taiwanese specimens include deeply divergent COI lineages without clear external morphological differences (Fikáček et al. 2023). Thus, newly collected East Asian sphaeriusids should not be assigned to named species based only on general body shape or DNA barcode similarity without broader integrative comparison. Before the present study, East Asian sphaeriusid records comprised genus‐level records from Japan and Taiwan (*Sphaerius* spp.), family‐level records from a handful of localities in Southern China, and a localized mainland record from Jiangxi, China ( *B. minutum* ) (Hall 2003; Kamezawa and Matsubara 2012; Liang and Jia 2018; Saito 2018, 2025; Fikáček et al. 2023). Mid‐Cretaceous Burmese amber documents greater past generic and morphological diversity, preserving the extant genera *Sphaerius* and *Bezesporum* alongside the extinct, morphologically distinctive †*Burmasporum* and †*Crowsonaerius* (Fikáček et al. 2023; Li et al. 2023).

Beyond Sphaeriusidae, other myxophagan records also suggest that suitable habitats in East Asia may harbor overlooked diversity. Torridincolidae, for example, are known from Japan and southeast China, including *Satonius kurosawai* from Japan and additional *Satonius* species from China (Satô 1982; Hájek and Fikáček 2008; Hájek et al. 2011). Members of this family are known to inhabit hygropetric habitats of pristine mountain waterfalls or seepages (Beutel and Vanin 2016), and in East Asia 13 species of *Satonius* Endrödy‐Younga, 1997 are known to date (Liang et al. 2024). In Japan, only *Satonius kurosawai* (M. Satô, 1982) is known from various localities in Honshu, Shikoku, and Yakushima Island (Nakajima et al. 2020), while in mainland China 12 species are described mainly from mountainous regions south of the Yangtze river (Liang et al. 2024).

In the Korean Peninsula, however, no member of Myxophaga has been recorded, despite national biodiversity inventories and recent taxonomic treatments mainly in South Korean aquatic Coleoptera (Bae et al. 2021). We therefore conducted targeted field surveys for Korean Myxophaga in potentially suitable riparian and hygropetric habitats, focusing especially on Sphaeriusidae and Torridincolidae.

During these surveys, we recovered Sphaeriusidae from two localities in the Naeseongcheon River basin. Here, we report these specimens as *Sphaerius* sp., representing the first documented record of Myxophaga from South Korea. This record extends the documented continental East Asian range of the suborder northeastward from previously published mainland Chinese records. We refrain from assigning a formal species name because the East Asian fauna of Sphaeriusidae remains taxonomically unresolved, many regional records are still unidentified or provisional, and reliable species delimitation will require broader integrative comparison of Korean, Chinese, Taiwanese, Japanese, and Southeast Asian populations (Kamezawa and Matsubara 2012; Liang and Jia 2018; Fikáček et al. 2023). We describe the riparian microhabitats where the specimens were collected and how these beetles were collected, and provide morphological evidence based on SEM photographs and COI barcode sequence data.

## 2 Materials and Methods

### 2.1 Collection Sites and Methods

Because the survey was specifically designed to target Myxophaga, candidate localities were selected based on the presence of microhabitats potentially suitable for the two focal families: wet riparian substrates along riverbanks and streams for Sphaeriusidae, and waterfall‐associated hygropetric habitats for Torridincolidae. Before fieldwork, potential sites were screened using online maps and recent web‐based information, including local webpages, Instagram posts, and YouTube videos, to confirm microhabitat suitability, accessibility, and field safety. The selected localities were then compiled and surveyed in the field (Table S1, Figure S1).

At each locality selected for Sphaeriusidae, searches focused on visibly wet or saturated substrates within approximately 1 m horizontally from the active waterline. Where present, large stones were first lifted or removed, and the surface layer of mixed sand, mud, fine gravel, small stones, and decomposing plant material was excavated. The collected substrate was placed in a large plastic storage container partly filled with water from the site and gently agitated. Floating material was inspected either directly on the water surface in the container or after being passed through a fine‐mesh sieve and transferred onto a white sorting tray for examination. On the other hand, waterfall‐associated hygropetric microhabitats, including wet rock faces, splash zones, seepages, mossy surfaces, and thin water films, were visually inspected for Torridincolidae, and beetles found on wet surfaces were collected.

### 2.2 Morphological Assessment

Collected specimens were transported to the laboratory and examined using an Olympus SZ61 stereomicroscope (Evident, Tokyo, Japan). Adult habitus photographs were taken using a Canon EOS R7 digital camera with a customized lens consisting of a Mitutoyo Mplan Apo 5× objective lens and a Raynox DCR‐150 close‐up lens. Image stacks were focus‐stacked using Helicon Focus software. For scanning electron microscopy (SEM), dried adult specimens were mounted on aluminum stubs using adhesive tape and sputter‐coated with gold using an ion sputter coater (G10). Specimens were examined and imaged using a COXEM EM‐30 scanning electron microscope.

### 2.3 Genetic Analyses

DNA extraction, COI amplification, sequencing, chromatogram editing, and alignment followed Lee et al. (2026). In brief, DNA was extracted from whole insects using the DNeasy Blood and Tissue Kit (QIAGEN) and an approximately 658‐bp fragment of mitochondrial cytochrome oxidase I (COI) was amplified using the COI primer sets described therein. PCR products were purified and Sanger‐sequenced by Bionics Inc. (Seoul, Korea). Sequence chromatograms were checked and assembled in SEQMAN PRO v.7.1.0 (DNASTAR Inc., Madison, WI, USA; Swindell and Plasterer 1997), and alignments were conducted in MEGA X (Kumar et al. 2018) under the amino acid translation option.

For phylogenetic comparison, we retrieved 19 available *Sphaerius* COI sequences from GenBank, with accession numbers listed in Table S2, and included two *Bezesporum* sequences. Because the concatenated five‐gene analysis and all single‐gene analyses except COI in Fikáček et al. (2023) recovered *Bezesporum* as sister to a monophyletic *Sphaerius*, we rooted our COI‐only tree using *Bezesporum* as the outgroup. The combined dataset of curated public sequences and newly generated sequences was analyzed in IQ‐TREE 2 under the Maximum‐likelihood framework (Minh et al. 2020). ModelFinder selected GTR + F + G4 as the best‐fit substitution model under the Bayesian Information Criterion (Kalyaanamoorthy et al. 2017). Nodal support was assessed using 1000 ultrafast bootstrap (UFBS) replicates (Minh et al. 2013; Hoang et al. 2018), and the resulting tree was visualized in FigTree v.1.4.4 (Rambaut 2018).

## 3 Results

### 3.1 Collection Sites and Habitat Description

**FIGURE 1:**
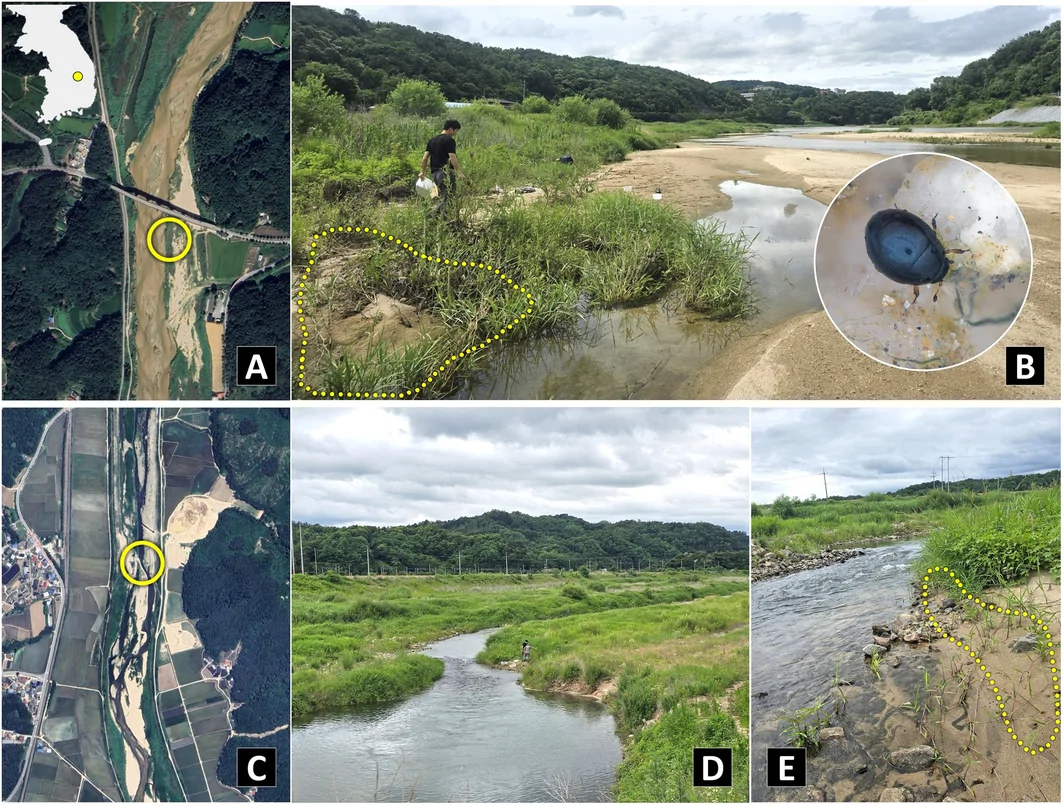
Collection localities and habitats of *Sphaerius* sp. in South Korea. (A, B) Locality A (A: Satellite image of the collection site; B: Riparian habitat at the site, with the sampled area indicated by a yellow dotted line and the circular inset showing an adult); (C–E) Locality B (C: Satellite image of the collection site; D: General view of the stream channel; E: Riparian microhabitat where adults were collected, with the sampled area indicated by a yellow dotted line).

Among all surveyed localities, *Sphaerius* was detected at only two sites. At locality 8 (Table S1, Figure 1A,B), a single adult was found after several hours of excavation and substrate sampling. Because substrate from several nearby points had been pooled during sampling, the exact microhabitat of this specimen could not be determined with certainty; however, it was most likely collected from the area indicated in Figure 1B or its immediate vicinity. After this discovery, the surrounding riparian substrate was searched intensively for approximately four additional hours using a larger shovel and container, but no further individuals were recovered. At locality 13 (Table S1, Figure 1C–E), *Sphaerius* was detected shortly after sampling began, and six adults were collected from several riparian microhabitats, including substrates immediately adjacent to the waterline. No *Sphaerius* specimens were detected at the remaining surveyed localities.

At localities selected for targeted searches of Torridincolidae, intensive visual inspection of hygropetric habitats yielded very few beetles overall. Hygropetric beetles like Psephenidae, Staphylinidae, and Hydrophilidae were observed in low density. Although other aquatic invertebrates, especially Blephariceridae larvae and Ephemeroptera nymphs, were frequently observed, beetles were rare, and no torridincolids were collected.

### 3.2 Morphological Examination Using Scanning Electron Microscope

**FIGURE 2:**
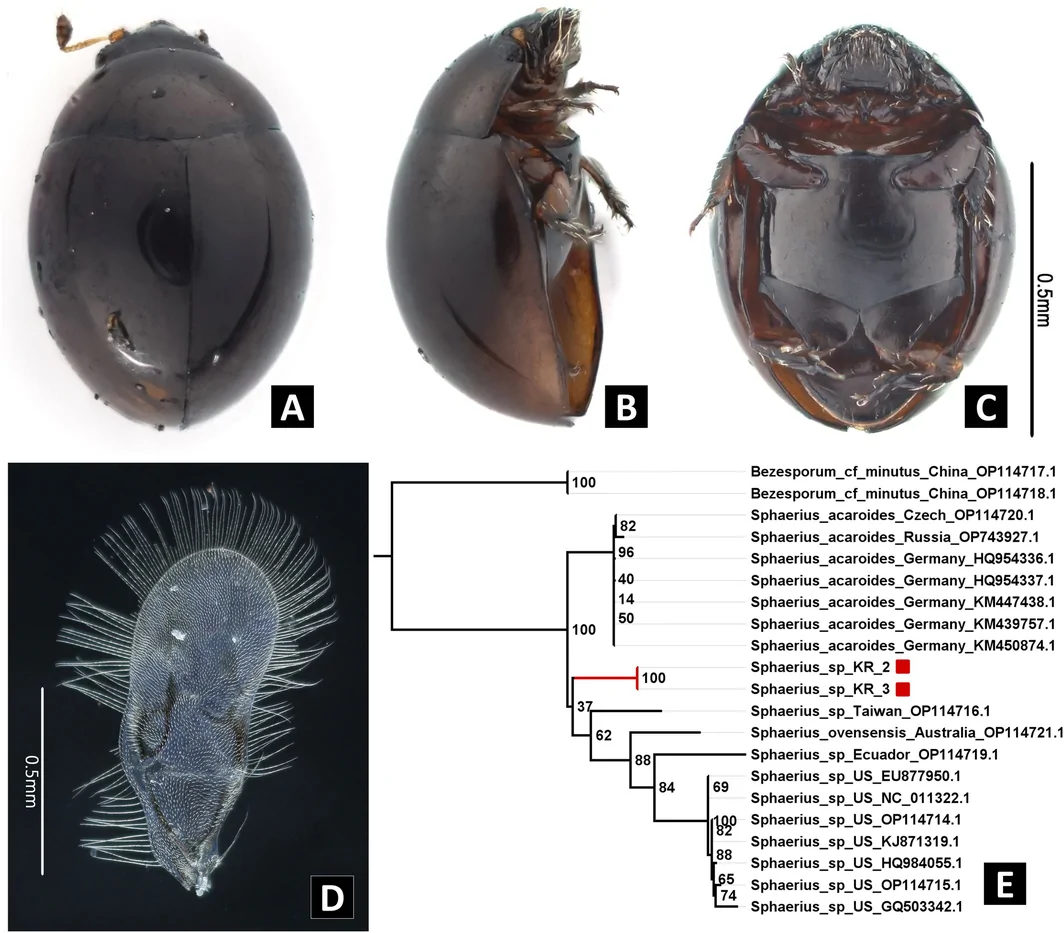
Habitus and COI phylogeny of Korean *Sphaerius* sp. (A–C) Adult habitus (A: Dorsal view; B: Lateral view; C: Ventral view); (D) Hind wing; (E) COI maximum‐likelihood tree inferred using IQ‐TREE, node values indicate bootstrap support.

**FIGURE 3:**
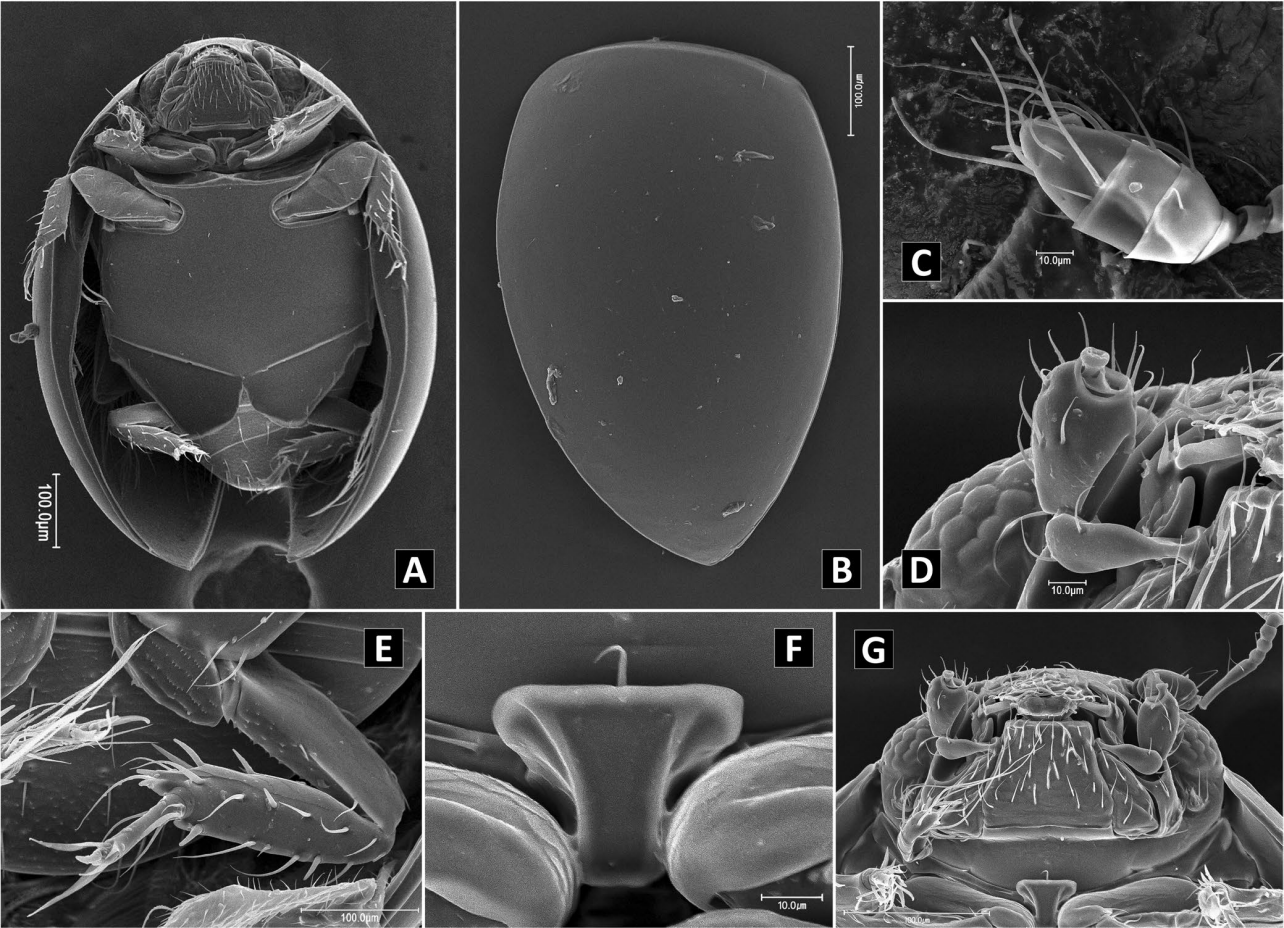
SEM micrographs of Korean *Sphaerius* sp. (A) Ventrum; (B) Elytron; (C) Antennal club; (D) Maxillary palpus; (E) Hind leg; (F) Prosternal elevation; (G) Ventral view of head.

Adults broadly hemispherical and strongly convex (Figure 2A–C). Head transverse, compound eyes well developed and strongly protruding. Clypeal carinae subparallel, mentum narrowing anteriad, with long setae (Figure 3G). Antennae 11‐segmented, with the terminal three or four antennomeres (see discussion) forming a distinct club and bearing long setae; antennomere VIII slightly swollen distally (Figure 3C). Maxillary palpus with enlarged palpomere followed by a minute distal palpomere (Figure 3D). Pronotum widest posteriorly, with the posterior margin slightly narrower than the base of the elytra. Prosternal elevation T‐shaped, posterior end moderately broad and not attenuated (Figure 3F). Mesoventral elevation wide and short, with a narrow rim along the anterior and lateral margins. Mesothoracic trochanter and femur fused into a trochantero‐femur, with the anterior margin straight (Figure 3A). Elytra smooth and strongly convex, without coarse punctures or sensorial fields (Figure 3B). Exposed abdominal ventrites three; terminal ventrite broadly rounded. Hind tarsi with at least two visible tarsomeres, although the total number could not be reliably determined (Figure 3E). Hindwings well developed, with strongly reduced venation, a distinct oblongum, and a complete fringe of long marginal hairs (Figure 2D).

### 3.3 DNA Barcoding and Phylogenetic Analysis

Two Korean specimens yielded complete 658‐bp sequences corresponding to the standard COI barcode region and were identical across the entire fragment. BLAST searches showed that the Korean sequences were most similar to available sequences of *Sphaerius acaroides* , with sequence identities of 91.79%–92.25%. The next closest match was *Sphaerius* sp. from Taichung, Taiwan, with a sequence identity of 90.43% (Figure S2).

In the phylogenetic analysis (Figure 2E), the Korean sequences were recovered as a single lineage, represented by two samples, labeled as Sphaerius_sp._KR_2 and Sphaerius_sp._KR_3 in the tree, with strong support (UFBS = 100). The European and Russian specimens of *Sphaerius acaroides* from Germany, the Czech Republic, and Russia formed a separate clade (UFBS = 96). This *S. acaroides* clade was placed apart from a second clade comprising the Korean lineage, the Taiwanese *Sphaerius* sp., *Sphaerius ovensensis* from Australia, and New World representatives from Ecuador and the United States. Within this second clade, the Korean lineage was recovered as sister to the assemblage containing the Taiwanese, Australasian, and New World taxa, although this relationship was weakly supported (UFBS = 37). The Taiwanese *Sphaerius* sp. was placed as sister to the Australasian–New World assemblage (UFBS = 62). Within the latter assemblage, *S. ovensensis* from Australia was recovered as sister to the Ecuadorian and North American lineages (UFBS = 88), and the Ecuadorian specimen was placed as sister to the North American *Sphaerius* sp. clade (UFBS = 84).

## 4 Discussion

The COI phylogeny reconstructed using IQ‐TREE suggests that the Korean specimens represent a distinct *Sphaerius* lineage among the sampled taxa. Although BLAST searches showed the highest similarity to *S. acaroides* , the phylogenetic analysis did not recover the Korean lineage within the European/Russian *S. acaroides* clade. Instead, the Korean lineage occupied a separate position near the base of a clade containing the Taiwanese, Australasian, and New World taxa, although this relationship was weakly supported. The topology therefore suggests possible geographic structuring among the sampled taxa, with the European/Russian *S. acaroides* clade separated from the Australasian–New World assemblage, while the Korean and Taiwanese lineages occupy separate East Asian positions in the COI tree. Topological differences between the multilocus analysis of Fikáček et al. (2023) and our COI‐only topology further emphasize the limited resolution of the present dataset.

The Korean specimens examined in this study are morphologically consistent with the “typical” *Sphaerius* morphotype sensu Fikáček et al. (2023) in having a mentum narrowing anteriad, a narrow prosternal elevation, and elytra lacking sensorial fields. The Korean specimens closely resemble Japanese and Taiwanese *Sphaerius*, with no conspicuous external differences visible in published SEM images (Kamezawa and Matsubara 2012; Fikáček et al. 2023). Their antennal clubs also appear essentially identical; the four‐ versus three‐segmented interpretations may reflect viewing angle and whether the slightly enlarged antennomere VIII is counted as part of the club. Given the morphological conservatism of *Sphaerius* (Fikáček et al. 2023), direct morphological and molecular comparisons, particularly with Japanese material, are needed to clarify the taxonomic affinities of these East Asian populations. Together with their COI similarity to *S. acaroides* and their phylogenetic position relative to the Taiwanese *Sphaerius*, this suggests that the Korean population should not be interpreted simply as an isolated faunistic occurrence. Rather, it may represent part of a poorly sampled East Asian component of typical *Sphaerius* diversity.

However, this interpretation remains preliminary because available sequence data are still limited for several key regions, particularly Taiwan, Japan, and China. The apparent relationship between the Korean and Taiwanese lineages may reflect incomplete taxon sampling, limited phylogenetic resolution of COI, or unsampled diversity in East Asia, and cannot be evaluated confidently with COI data alone. Thus, denser geographic sampling from Korea, Taiwan, Japan, and China, additional nuclear markers, multilocus phylogenetic analyses, and SEM‐based examination of voucher specimens will be essential. In this context, the present study should be regarded as a stepping stone toward a more comprehensive and robust taxonomic revision of East Asian *Sphaerius*.

Although the currently known East Asian records may appear disjunct (Table S3), this pattern likely reflects insufficient targeted sampling. In Japan, *Sphaerius* has been recorded from several regions, and Kamezawa and Matsubara (2012) suggested that additional localities would probably be discovered through careful investigation. Fikáček et al. (2023) similarly emphasized that the distribution and diversity of Sphaeriusidae remain underestimated because of their minute body size, cryptic habits, and specific collecting requirements. The Korean occurrence should therefore be interpreted not as an isolated distributional anomaly, but as evidence that the regional connectivity and diversity of East Asian *Sphaerius* remain poorly understood.

This discovery also highlights the importance of microhabitat‐focused sampling in Korea. Although national biodiversity surveys have expanded and accelerated taxonomic studies in Korea (Bae et al. 2021), minute and habitat‐specific beetles such as Sphaeriusidae are easily overlooked by general collecting methods. The Korean sites closely resemble habitats reported from Japan and China, as well as those summarized by Fikáček et al. (2023), including moist gravel, saturated shore sediment, stones at stream margins, and fine sediment near the waterline. Consistent with the collecting methods summarized by Fikáček et al. (2023), flotation of the upper layer of shore sediment also proved effective in South Korea. Numerous Hydrophilidae, Heteroceridae, Carabidae, and Staphylinidae were observed during our surveys, suggesting that the apparent rarity of *Sphaerius* does not simply reflect a general depletion of the riparian beetle fauna, although species‐specific effects of habitat degradation cannot be excluded.

Future surveys in South Korea should target a broader range of wet riparian microhabitats, including lowland rivers, upper stream reaches, gravel bars, lake margins, stony creeks, irrigation channels, and rice‐field margins. Co‐occurrence with other shore‐associated microcoleopterans may provide a useful field cue, as Sphaeriusidae are often collected together with Georissidae, some Hydrophilidae, Epimetopidae, Limnichidae, Hydraenidae, and other riparian insects (Fikáček et al. 2023; Saito 2025). The recent first Korean record of Georissidae from a mountain stream in Gangwon‐do (Lee et al. 2024) further suggests that comparable stream‐margin habitats in Korea deserve attention. More broadly, the discovery of Korean *Sphaerius* indicates that other overlooked minute beetle lineages may remain undetected, and that microhabitat‐focused collecting is essential for a more comprehensive understanding of Korean and East Asian beetle diversity.

## Author Contributions

**Seunghyun Lee:** conceptualization (equal), data curation (equal), formal analysis (equal), funding acquisition (equal), investigation (equal), methodology (equal), project administration (lead), resources (lead), validation (lead), visualization (equal), writing – original draft (lead), writing – review and editing (equal). **Minhyeuk Lee:** conceptualization (equal), data curation (equal), funding acquisition (equal), investigation (equal), methodology (equal), visualization (equal), writing – review and editing (equal). **Wonwoong Kim:** data curation (equal), investigation (equal), visualization (equal), writing – review and editing (equal).

## Funding

National Research Foundation of Korea (NRF), Grant/Award Numbers: RS‐2024‐00405751 and RS‐2024‐00452022. National Institute of Agricultural Sciences, Rural Development Administration, Republic of Korea (Project No. PJ017645).

## Conflicts of Interest

The authors declare no conflicts of interest.

## Supporting information


**Figure S1:** Surveyed localities in this study.

**Figure S2:** BLAST results based on the COI sequences obtained in this study.

**Table S1:** Surveyed localities in this study.

**Table S2:** Sequences used in this study and their GenBank accession numbers.

**Table S3:** Published records of Sphaeriusidae from East Asia.

## Data Availability

The data that support the findings of this study are openly available in Figshare (https://doi.org/10.6084/m9.figshare.32857958).

## References

[ece374411-bib-0001] Bae, Y. J. , K. Cho , G.‐S. Min , et al. 2021. “Review of the Korean Indigenous Species Investigation Project (2006–2020) by the National Institute of Biological Resources Under the Ministry of Environment, Republic of Korea.” Korean Journal of Environmental Biology 39, no. 1: 119–135. 10.11626/KJEB.2021.39.1.119.

[ece374411-bib-0002] Beutel, R. G. 2005. “Myxophaga.” In Handbook of Zoology, Volume IV, Arthropoda: Insecta, Part 38, Coleoptera, Beetles, Vol. 1: Morphology and Systematics (Archostemata, Adephaga, Myxophaga, Polyphaga Partim), edited by R. G. Beutel and R. A. B. Leschen , 43–52. De Gruyter.

[ece374411-bib-0003] Beutel, R. G. , and R. Arce‐Pérez . 2016. “Sphaeriusidae Erichson, 1845 (Jäch 1999) (= Microsporidae).” In Handbook of Zoology, Vol. IV Arthropoda: Insecta. Part 38. Coleoptera, Vol. 1: Morphology and Systematics (Archostemata, Adephaga, Myxophaga, Polyphaga Partim), edited by R. G. Beutel and R. A. B. Leschen , 70–71. Walter de Gruyter.

[ece374411-bib-0004] Beutel, R. G. , and S. A. Vanin . 2016. “Torridincolidae Steffan, 1964.” In Handbook of Zoology. A Natural History of the Phyla of the Animal Kingdom. Vol. IV. Arthropoda: Insecta. Part 38. Coleoptera, Beetles. Vol. 1. Morphology and Systematics (Archostemata, Adephaga, Myxophaga, Polyphaga Partim.), edited by R. G. Beutel and R. A. B. Leschen , 2nd ed., 67–70. Walter de Gruyter.

[ece374411-bib-0005] Fikáček, M. , S. Yamamoto , K. Matsumoto , R. G. Beutel , and D. R. Maddison . 2023. “Phylogeny and Systematics of Sphaeriusidae (Coleoptera: Myxophaga): Minute Living Fossils With Underestimated Past and Present‐Day Diversity.” Systematic Entomology 48, no. 2: 233–249. 10.1111/syen.12571.

[ece374411-bib-0006] Hájek, J. , and M. Fikáček . 2008. “A Review of the Genus *Satonius* (Coleoptera: Myxophaga: Torridincolidae): Taxonomic Revision, Larval Morphology, Notes on Wing Polymorphism, and Phylogenetic Implications.” Acta Entomologica Musei Nationalis Pragae 48: 655–676.

[ece374411-bib-0007] Hájek, J. , H. Yoshitomi , M. Fikáček , M. Hayashi , and F.‐L. Jia . 2011. “Two New Species of *Satonius* Endrödy‐Younga From China and Notes on the Wing Polymorphism of *S. kurosawai* Satô (Coleoptera: Myxophaga: Torridincolidae).” Zootaxa 3016: 51–62. 10.11646/zootaxa.3016.1.4.

[ece374411-bib-0008] Hall, W. E. 2003. “Sphaeriusidae (Coleoptera).” In Water Beetles of China. Volume III, edited by M. A. Jäch and L. Ji , 37–41. Zoologisch‐Botanische Gesellschaft and Wiener Coleopterologenverein.

[ece374411-bib-0009] Hall, W. E. 2019. “Sphaeriusidae.” In Australian Beetles. Volume 2: Archostemata, Myxophaga, Adephaga, Polyphaga (Part), edited by A. Ślipiński and J. Lawrence , 15–17. CSIRO Publishing.

[ece374411-bib-0010] Hoang, D. T. , O. Chernomor , A. von Haeseler , B. Q. Minh , and L. S. Vinh . 2018. “UFBoot2: Improving the Ultrafast Bootstrap Approximation.” Molecular Biology and Evolution 35: 518–522. 10.1093/molbev/msx281. PMC5850222 29077904

[ece374411-bib-0011] Kalyaanamoorthy, S. , B. Q. Minh , T. K. F. Wong , A. von Haeseler , and L. S. Jermiin . 2017. “ModelFinder: Fast Model Selection for Accurate Phylogenetic Estimates.” Nature Methods 14: 587–589. 10.1038/nmeth.4285. PMC5453245 28481363

[ece374411-bib-0012] Kamezawa, H. , and Y. Matsubara . 2012. “A Faunistic Note on a Species of the Genus *Sphaerius* Waltl, 1838 (Myxophaga, Sphaeriusidae) Collected From Tamagawa River, Tokyo Metropolis, Central Honshu, Japan.” Sayabane, New Series 6: 25–27.

[ece374411-bib-0013] Kumar, S. , G. Stecher , M. Li , C. Knyaz , and K. Tamura . 2018. “MEGA X: Molecular Evolutionary Genetics Analysis Across Computing Platforms.” Molecular Biology and Evolution 35: 1547–1549. 10.1093/molbev/msy096. PMC5967553 29722887

[ece374411-bib-0015] Lee, D.‐H. , S. W. Jung , Y.‐H. Kim , and I. C. Shin . 2024. “First Record of the Beetle Family Georissidae (Coleoptera) in Korea, With a mtDNA Information of a Species.” Journal of Species Research 13, no. 3: 326–328. 10.12651/JSR.2024.13.3.326.

[ece374411-bib-0016] Lee, S. , S. Sul , and S. Lee . 2026. “Molecular Identification of Immature Longhorn Beetles (Cerambycidae and Disteniidae) From South Korea.” Journal of Zoological Systematics and Evolutionary Research 2026: 1583929. 10.1155/jzs/1583929.

[ece374411-bib-0017] Li, Y.‐D. , A. Ślipiński , D.‐Y. Huang , and C.‐Y. Cai . 2023. “New Fossils of Sphaeriusidae From Mid‐Cretaceous Burmese Amber Revealed by Confocal Microscopy (Coleoptera: Myxophaga).” Frontiers in Earth Science 10: 901573. 10.3389/feart.2022.901573.

[ece374411-bib-0018] Liang, Z.‐L. , and F. Jia . 2018. “A New Species of *Sphaerius* Waltl From China (Coleoptera, Myxophaga, Sphaeriusidae).” ZooKeys 808: 115–121. 10.3897/zookeys.808.30600. PMC6305768 30598609

[ece374411-bib-0019] Liang, Z.‐L. , Z.‐Y. Jiang , J. Bergsten , F.‐L. Jia , and S.‐Q. Ge . 2024. “A Review of the Genus *Satonius* Endrödy‐Younga From China With Description of Seven New Species (Coleoptera: Myxophaga: Torridincolidae).” Zootaxa 5528, no. 1: 89–105. 10.11646/zootaxa.5528.1.8. 39646900

[ece374411-bib-0020] Löbl, I. 1995. “New Species of Terrestrial *Microsporus* From the Himalaya (Coleoptera: Microsporidae).” Entomologische Blätter 91: 129–138.

[ece374411-bib-0021] Matthews, A. 1899. A Monograph of the Coleopterous Families Corylophidae and Sphaeriidae. O. E. Janson & Son.

[ece374411-bib-0022] Minh, B. Q. , M. A. T. Nguyen , and A. von Haeseler . 2013. “Ultrafast Approximation for Phylogenetic Bootstrap.” Molecular Biology and Evolution 30: 1188–1195. 10.1093/molbev/mst024. PMC3670741 23418397

[ece374411-bib-0023] Minh, B. Q. , H. A. Schmidt , O. Chernomor , et al. 2020. “IQ‐TREE 2: New Models and Efficient Methods for Phylogenetic Inference in the Genomic Era.” Molecular Biology and Evolution 37: 1530–1534. 10.1093/molbev/msaa015. PMC7182206 32011700

[ece374411-bib-0024] Nakajima, J. , M. Hayashi , K. Ishida , T. Kitano , and H. Yoshitomi . 2020. 日本の水生昆虫 [Aquatic Coleoptera and Hemiptera of Japan]. Bun‐ichi Sogo Shuppan [In Japanese].

[ece374411-bib-0025] Qvarnström, M. , M. Fikáček , J. V. Wernström , et al. 2021. “Exceptionally Preserved Beetles in a Triassic Coprolite of Putative Dinosauriform Origin.” Current Biology 31, no. 15: 3374–3381. 10.1016/j.cub.2021.05.015. 34197727

[ece374411-bib-0026] Rambaut, A. 2018. “FigTree v1.4.4.” Institute of Evolutionary Biology, University of Edinburgh.

[ece374411-bib-0027] Reichardt, H. 1973. “A Critical Study of the Suborder Myxophaga, With a Taxonomic Revision of the Brazilian Torridincolidae and Hydroscaphidae (Coleoptera).” Arquivos de Zoologia 24: 73–162. 10.11606/issn.2176-7793.v24i2p73-162.

[ece374411-bib-0028] Saito, T. 2018. “ケシマルムシ属微小甲虫を神奈川県で採集 [A Minute Beetle of the Genus *Sphaerius* Collected in Kanagawa Prefecture].” Sayabane New Series 31: 26–27 [In Japanese].

[ece374411-bib-0029] Saito, T. 2025. “神奈川県におけるシワムネマルドロムシとケシマルムシ属の一種の生態観察例 [Ecological Observations of *Georissus Kurosawai* and a Species of *Sphaerius* in Kanagawa Prefecture].” Sayabane New Series 59: 72–74 [In Japanese].

[ece374411-bib-0030] Satô, M. 1982. “Discovery of Torridincolidae (Coleoptera) in Japan.” Annotationes Zoologicae Japonenses 55: 276–283.

[ece374411-bib-0031] Swindell, S. R. , and T. N. Plasterer . 1997. “SEQMAN.” In Sequence Data Analysis Guidebook, edited by S. R. Swindell , vol. 70, 75–89. Humana Press. 10.1385/0-89603-358-9:75.

[ece374411-bib-0032] Waltl, J. 1838. “Verzeichniss Der um Passau Vorkommenden Seltener Käfer Nebst Beschreibung Der Neuen Arten.” Isis Von Oken 4: 263–273.

